# Developing a framework for understanding health information behavior change from avoidance to acquisition: a grounded theory exploration

**DOI:** 10.1186/s12889-022-13522-0

**Published:** 2022-06-04

**Authors:** Haixia Sun, Jiao Li, Ying Cheng, Xuelian Pan, Liu Shen, Weina Hua

**Affiliations:** 1 Institute of Medical Information & Library, Chinese Academy of Medical Sciences / Peking Union Medical College, Beijing, China; 2 School of Information Management, Nanjing University, Nanjing, China

**Keywords:** Public health informatics, Information avoidance, Consumer health information, Information services, Health behavior, Grounded theory

## Abstract

**Background:**

Health information avoidance is common in real life, but because it is not always conducive to health promotion and maintenance, people often actively switch to health information acquisition. Understanding this process of active change can facilitate intervention in unreasonable avoidance behaviors. However, studies so far have mostly focused on why and how avoidance takes place, little is known about the process of active change from avoidance to acquisition. We thus use a grounded theory approach (GT) to explore how the active change takes place, and to generate a grounded theoretical framework capable of illustrating stages and influencing factors involved in the active change process.

**Methods:**

Straussian grounded theory (Corbin & Strauss, 2015) was used to analyze data collected through semi-structured interviews with 30 adults (14 in good health, 11 with disease, 5 in other health status) who had experienced health information behavior change from avoidance to acquisition. These interviews focused on how the change occurred and what effected the change.

**Results:**

The core category of Health Information Avoidance Change and 12 categories were identified and integrated to form a theoretical framework termed the Health Information Avoidance Change Model (HIACM). This model describes the process using five non-linear stage variables (initiation, preparation, action, maintenance, and abandonment) and seven moderating factor variables (cognitive change, social stimulus, beliefs and attitudes, intrapsychic literacy, social resources, information source, time and material resources).

**Conclusions:**

HIACM can be used to explain the process of active change from health information avoidance to health information acquisition. HIAC is a non-linear and holistic process, and it is necessary to dynamically analyze the impact of relevant factors and take targeted intervention measures in stages. HIAC is usually not only an individual behavior, but also a socialized behavior requiring the collaboration of individuals, families, health information providers, healthcare providers, and governments.

**Supplementary Information:**

The online version contains supplementary material available at 10.1186/s12889-022-13522-0.

## Background

Despite the importance of learning about one’s health issues, risks, or promotion, empirical evidence suggests that people may actively avoid related health information completely or selectively in daily health maintenance and promotion contexts [1–6], such as genetic testing [7, 8], cancer screening [9], general physical examination [10], medication use [11], and red meat risks [12]. For example, 21% of college women and 24% of women aged 35 and older opted not to learn their breast cancer risk [13], 29% of Americans indicate that they would avoid visiting the doctor despite suspecting they should [14], and 34.2% of college students agree or strongly agree with such statements as “I would rather not know the extent of sun damage to my skin” [15]. Even in the context of an event such as the coronavirus epidemic, which is relevant to everyone, many people choose to avoid related topics such as prevention and mortality [16–18]. These findings show information avoidance to be a relatively common type of information behavior in the context of health.

Information avoidance is defined as “any behavior intended to prevent or delay the acquisition of available but potentially unwanted information” [4], although the individuals involved do not know the specific content of this information [19]. Previous studies have shown that in the short term, health information avoidance satisfies the individual’s hedonic needs [5]; the dark side is that it not only affects individuals’ attitudes toward health risk information [12], health self-evaluation [20], medical screening [14], and disease treatment [21], but also undermines the prevention and control of public health problems across society in the context of infectious diseases [17, 22, 23]. Therefore, there is a need to understand health information avoidance and explore behavioral interventions that may reduce unreasonable information avoidance.

Many studies have been conducted on the motivation and influencing factors of health information avoidance (HIA). It has been shown that HIA is a conscious or unintentional defensive response when individuals perceive the threats that information may bring [24, 25]. The motivations for this response mainly involves a mixture of emotions, cognition, and behavior: 1) to maintain pleasant emotions or avoid negative emotions, such as fear, anxiety, worry, or distress. [16, 17, 22]; 2) to avoid cognitive dissonance [11]; and 3) to avoid unwanted actions (e.g., sun-protective behavior [15] or the imposition of limitations on their actions [6, 26].

Influencing factor analyses show that HIA is primarily related to sociodemographic characteristics, coping resources, health information characteristics, and situations. Sociodemographic characteristics include gender, age, education level, medical insurance, self-rated health status, etc. [7, 9, 20, 21, 27–29]. Coping resources are those resources that individuals possess or can obtain from outside to deal with health information threats [9, 21, 25, 29–36], including beliefs and attitudes, self-efficacy, social support, personality traits, and health information literacy; these are negatively related to HIA. Health information characteristics [7, 9, 13, 16, 20, 28, 30, 32, 37] mainly refer to the form of information dissemination, along with information quality and information overload. If these characteristics bring about negative judgments such as difficulty in access and understanding, cognitive overload, unreliability, and impracticality, individuals tend to avoid health information. Situational factors [27, 28, 30, 34, 38, 39] include macro-level public health policies and medical technology, as well as micro-level locations and audiences. Public health policies determine the coping resources that individuals can obtain from society, thereby influencing HIA. The state of medical technology determines whether a disease is curable or preventable, which is also negatively related to the avoidance of specific disease information.

A few prior studies have engaged with HIA interventions based on self-enhancement theory and the above findings concerning avoidance motivation and influencing factors: for example, through self-affirmation and self-enhancement intervention to weaken the perception of health information threats [40], and moderate the avoider’s psychological defenses response to health risk information [41, 42]. For the most part, these are experimental verification studies that (to an extent) assume health information avoidance behavior change to be a single event and emphasize the result of such change while overlooking its processual nature. However, according to theories of healthy behavior change such as the transtheoretical model (TTM), individual behavior change is a complex developmental process, and this process merits greater attention than the result [43]. TTM [43, 44] states that the change involves five or six stages (precontemplation, contemplation, preparation, action, maintenance, and termination), and to some extent maintains that the change process is sequential [45]. Previous information behavior studies, however, show that the information behavior processes are non-linear and heuristic [46–48]. Therefore, we assume that TTM may not be effective in illustrating HIA interventions; more detailed analysis is necessary to understand the process of change from health information avoidance to acquisition.

Some information science theories have suggested that information acquisition or seeking has multiple stages, each characterized by different activity and thoughts [44, 49]. However, considering the defensive psychology and behavior toward health information before the change, we assume that the change from health information avoidance to health information acquisition can not simply be equated with general health information acquisition or seeking, though there is overlap; hence the need for further investigation to understand how the “changer” changes from avoiding to acquiring health information, so that practitioners can dynamically intervene in health information avoidance behavior stage-by-stage.

Therefore, we aim to develop an empirical-theoretical framework to reflect the stages of the change, possible relationships among stages, and influencing factors. The grounded theory approach is process-oriented, enabling development of substantive theories that explain human interaction [50], and is thus appropriate for this purpose.

## Methods

### Aim

The goal of this study is to generate an empirical-theoretical framework for explaining, from a process-level perspective, how people change from health information avoidance to acquisition. The specific aims are to (1) identify stages and related activities during the change; (2) identify factors associated with the change; and (3) relate these stages and factors in an integrated theoretical framework that deeply illustrates the process of change.

### Study design

This study is conducted using Straussian grounded theory (SGT) [51], one of three main approaches to grounded theory (GT), which focuses on the theorization of latent behavioral patterns and generation of a theory capable of guiding practical action for problem solving [52]. Unlike classic grounded theory and constructive grounded theory (the other two major approaches), SGT suggests appropriate use of literature throughout all research phases and offers a codification paradigm to guide systematic coding, help establish relationships between categories, and identify the central category of the research [53]. Although there is no theoretical model directly related to the issue under study here, the preexisting research results related to HIA, models in information behavior [54], and behavior change can provide important theoretical references for data collection and analysis. SGT is therefore considered the ideal method for this study.

### Ethical considerations

This study was approved by the Ethics Committee at the Institute of Medical Information & Library, Chinese Academy of Medical Sciences (statement no. IMICAMS/03/20/HREC). Participants were informed of the study details prior to the interview, and all participants provided their signed, written informed consent.

### Sampling and recruitment

Individuals were eligible to participate if they: 1) were at least 18 years of age; 2) were able to understand our questions and clearly express their responses in Mandarin Chinese; 3) had a substantive experience of behavior change from avoiding health information to acquiring health information (not just the intention to change); and 4) were volunteering to participate in this research.

Theoretical sampling (TS) [55] was used to select potential participants throughout the data collection. TS refers to iterative data collection and analysis, allowing for subsequent data collection to be informed by the specific need to deepen knowledge according to analysis of the previous data collected. Sociodemographic and health status related to health information avoidance in existing research were used to guide the selection of samples. Strategies for HIA found in existing research were used to identify participants with experiences of avoiding health information, such as physical avoidance (e.g., avoiding going to the doctor [56], cognitive avoidance (e.g., controlling conversation [26], denial and cognitive reappraisal [57] and language avoidance (e.g., “not wanting to inform others” [39] and “I don't know” responses in the questionnaire [58].

TS was undertaken in 3 stages: open sampling, variation sampling, and differential sampling [51]. The open sampling began with people considered most likely to answer our research question and achieve the research objectives. Previous descriptive statistics implied that young healthy people and people with incurable disease or prior experience with serious illness were more inclined to avoid health information [2, 26]. Therefore, in this stage we sought participants with the following characteristics: 1) under 45 years old and in good health; or 2) cancer survivors. In the second stage, samples that might demonstrate different properties of concepts and show variation were selected. We mainly sought variant samples based on age, health status, education, and occupation, such as the elderly, people with chronic diseases, retirees, and medical staff. Differential sampling, focused mainly on differences in participants’ marital status and geographical location, was conducted to develop unsaturated categories and verify the theoretical framework generated in stages 1 and 2 by achieving data saturation.

Purposive sampling (selection of participants who met the inclusion criteria) and convenience sampling (recruitment of potential participants via invitation letters through interpersonal relationships and referral to the researchers’ colleagues, classmates and previous project partners) were both employed in the open sampling stage. Snowball sampling, i.e., inviting participants to refer other potential participants, was used later. Specifically, the first author began by introducing the background, objectives, and sample inclusion criteria of this research to the recommenders. Then, at different sampling stages, recommenders were asked to scan their surroundings for potential samples that met the theoretical sampling requirements of the corresponding stage. When a prospective match was found, the first author or the recommenders sent the invitation letter, depending on the willingness of the potential participant. The researcher and the recommenders verbally solicited the potential participant’s consent in advance, and informed them that even if they accepted the invitation letter, they could still refuse to participate in the research.

Two individuals with high education level (undergraduate level or above) refused the interview, citing “cannot say it clearly” and “do not want to say” as their reasons, after accepting the invitation letter forwarded by the recommenders. We determined this was largely related to privacy attitudes. A medical staff member who had accepted the invitation cancelled the interview because of time conflict; however, two other medical staff participated in the interview in the follow-up, so we thought this had little effect on the analysis results. The study rejected 4 potential interviewees (who were recommended but not contacted) because their sociodemographic and health status were highly similar to those of previous interviewees in the differential sampling stage.

### Data collection

Data collection and analysis were interrelated and simultaneous. Semi-structured interviews were undertaken from October 2019 to May 2020. Each interview lasted approximately 40–70 min. When ambiguities arose in the process of data analysis, clarification was sought by inviting the interviewee to accept a second interview.

A semi-structured interview guide (see Additional file 1) was developed based on the above specific objectives. With ongoing data collection and analysis, additional, more specific questions were developed to compare the emerging phenomena and concepts, supported by the review of literature related to HIA, health information acquisition, and behavior change. For example, when Participant 10 (male, corporate employee, undergraduate degree) explained the reasons for ending his change by simply mentioning lack of time, the following specific question was asked, inspired by the finding that the quality of health information may influence health information avoidance behaviors [26]: “*Apart from lack of time, was there any other reason that influenced your decision? For example, the provided information was not practical*”.

The interviews were carried out by the first author, an academic and practitioner focusing on promoting health information organizations and services, and who has personally experienced the change from health information avoidance to acquisition, which helped her gain the trust of the interviewees and encouraged them to share their own experiences. The others authors were accountable for ensuring that questions related to the accuracy and integrity of the research were appropriately investigated. Each participant engaged in one audio-recorded semi-structured interview via telephone, WeChat, or face-to-face in a private room, as preferred by the participant.

A single interview included 4 steps: 1) The researcher introduced the study, and an informed consent form was signed (if not completed in advance) to address the interviewee’s privacy concerns. The participant had an opportunity to ask questions and to opt out of the interview. 2) Questions and exchanges followed. To depict the situation as authentically as possible, we also noted nonverbal emotional reactions. 3) The interviewee completed a sociodemographic and health questionnaire (see Additional file 2). Questionnaire data were used to support subsequent sampling. 4) The interviewee was invited to recommend heterogeneous samples if possible.

### Data analysis

Sociodemographic data were summarized. Analysis of the qualitative data began almost immediately as data were collected, following GT principles. Constant comparative analysis was carried out to help generate theory, meaning continuous comparisons between the data, codes, and categories for the purpose of developing a greater understanding of the data. This process guided theoretical sampling and evolving questions asked of participants. Memo writing occurred throughout data collection and analysis. To avoid subjective bias, strategic reflexivity (writing reflexive notes), contextual-discursive reflexivity (continuously examining the situational elements in the data, such as needs, information, and time), relational reflexivity (continuously examining whether the researcher separated what belongs to the researcher from what pertains to the interviewee’s) [59] and collaborative coding (first back-to-back, then concentrated discussion) were used throughout the research process. Previous theoretical knowledge and sensitizing concepts were utilized to construct empirically grounded categories via a review of literature. NVivo (Version 12 Plus), a computer-assisted qualitative data analysis tool, was used to facilitate data management and analysis.

**Fig. 1:**
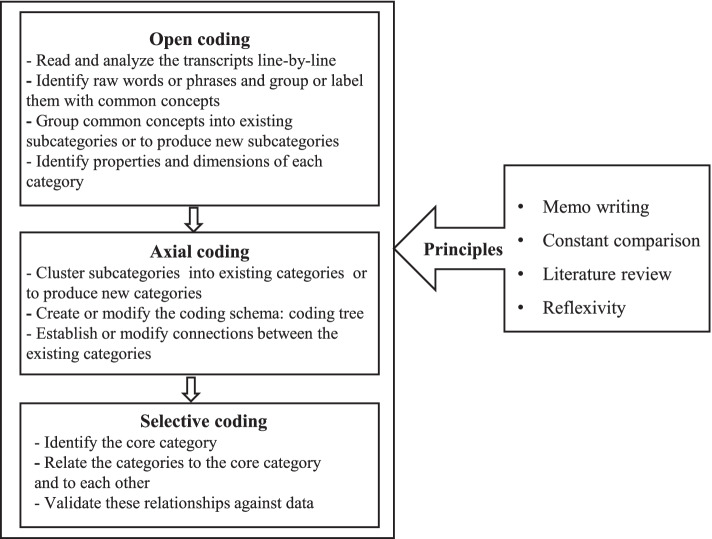
Steps of data analysis in accordance with SGT method

Open, axial, and selective coding of SGT analysis were undertaken [51] (see Fig. 1), initially by researchers HXS and LS, then including JL and XLP. YC and WNH guided the coding process and participated in the discussion to solve discrepancies. In the open coding stage, the researchers read and analyzed the transcripts line-by-line in search of raw words or phrases that expressed the essential meaning of the interviewees’ discourse and grouped them with common concepts. Related concepts were then grouped to produce subcategories. Names and definitions were given to each subcategory; properties and dimensions of each category were identified. To maintain the consistency of the concepts, existing and widely used concept names (e.g., self-efficacy [60] and health information literacy [61]) are used as much as possible to represent their accepted meanings.

In the axial coding stage, subcategories were clustered into categories and a coding schema was created. This schema was then used to guide further data collection and modified in subsequent analysis. The paradigm analytic tool and its three features (conditions, actions–interactions and consequences) were used to helped with category development and establish or modify connections between the existing categories to form an initial framework [51]. Where newly acquired data did not fit the framework or required a different code to explain its meaning, a new subcategory or category was created and integrated into the framework.

Selective coding was performed to refine the core category to be sufficiently broad and abstract, summarizing in a few words the main ideas expressed by all of the data in this study, and linking all categories together to form a theoretical model. This process took place by clarifying the story line, relating the categories to each other and to the emerging core category, validating these relationships against data, and finally filling in the categories that might need further refinement through theoretical sampling.

### Saturation

Data collection ceased when theoretical saturation occurred, where by no new concepts emerged from ensuing interviews. Data analysis began after the first data was obtained, and each subsequent interview was used to fill in poorly developed categories and revise the framework until no new concepts and relationships emerged. Theoretical sensitivity assisted in achieving saturation. Data saturated when the analysis of the 28th interview was completed; two further interviews were then undertaken to confirm this was indeed the case through constant comparative analysis.

### Rigor

Several steps or strategies were undertaken to promote credibility and validity throughout the study process. Theoretical sampling dominated the selection of samples to ensure the external validity of the research conclusions to the greatest extent. The internal validity of the constructed theory is guaranteed by the guiding principles of grounded theory methodology. Timely memo writing was undertaken to ensure that analysis results could be tracked for review. Consistency was enhanced by providing adequate explanations of all subcategories and categories. Continuous literature review and team meetings on the ongoing analysis and the upcoming data collection were conducted, which helped to ensure theoretical sensitivity. Reflection strategies were used to avoid analytical bias. The accuracy of naming and definitions was also ensured by consulting external experts (informatics, public health promotion and GT method). Eight participants (undergraduate and postgraduate degree) agreed to verify the suitability and explanatory power of the final theoretical model. All accepted it and no amendments were proposed.

Data accuracy was maintained by thoroughly studying the transcription of interviews. The clarity and consistency of interviewees’ presentations were improved by repeating questions, completing the transcription of a new audio files within 3 days of the interview, and inviting interviewees to proofread the transcripts.

## Results

A total of 30 adults participated in our interview. None of the interviewees withdrew midway. Seven interviewees participated in the second interview because of vague and conflicting descriptions in their first interview. Table 1 shows the sociodemographic profile of the 30 participants sampled. The sample was relatively balanced in terms of health status, with ages ranging from under 20 to the early 70 s. Importantly, except for participants under the age of 20, who were all college students in good health, samples of each age group cover different health status, education level, and occupation. 7 participants had chronic diseases: diabetes (3), hypertension (2), chronic bronchitis (2); 3 participants had major diseases at the time of the study: breast cancer (2), chronic renal failure uremic stage (1); and 1 participant had suffered from acute stroke.

**Table 1:** Participants’ sociodemographic profiles (*n* = 30)

Variable	Value	Variable	Value
**Gender**		**Education Level**	
Male	9	No formal education	1
Female	21	High school and below	6
**Age (Years)**		Junior college	6
18–25	3	Undergraduate	10
26–45	15	Postgraduate	7
46–60	9	**Occupation**	
≥ 61	3	College student	3
**Marital Status**		Corporate employee	10
Unmarried	8	Medical staff	2
Married	20	Civil servant	2
Divorced	1	Researcher	3
Widowed	1	Retiree	7
**Health Status**		Farmer	3
In good health	14	**Region**	
With chronic disease	7	Urban	27
With major disease	3	Rural	3
Previous major disease	1		
Other	5		

12 categories and 36 subcategories emerged from the data (see Table 2) that reflect the stages and activities during the change and factors moderating the dynamic interactions among stages. An additional codebook (see Additional file 3) shows all subcategories’ names, examples of open concepts, definitions and categories in more detail. The core category, which describes the behavioral phenomenon of interest and links the categories together, is Health Information Avoidance Change (HIAC): an intentional response to reduce or eliminate health information acquisition avoidance behavior.

**Table 2:** HIAC coding variables: stages, factors, and associated subcategories

**Stages**	**Factors**
*Initiation*	*Social stimuli*
Physical needs	Role changes
Cognitive needs	Social norms
Emotional needs	Key events
Social needs	
*Preparation*	*Cognitive change*
Planning time	Expanded health knowledge
Preparing materials	Change in perception of barrier
Seeking channels	Change in perceived severity
Evaluation	Change in perceived susceptibility
*Action *	*Beliefs and attitudes*
Active seeking	Health beliefs
Passive acquisition	Health information beliefs
Proxy seeking	Health information behavior beliefs
	Satisfaction
	Privacy attitudes
*Maintenance*	*Intrapsychic resources*
Self-regulation	Self-efficacy
Information focus	Health information literacy
*Abandonment*	*Social resources*
Weaken acquisition	Social support
Stop acquisition	
Health information avoidance	
	*Information source*
	Complexity of access
	Information quality
	Privacy policy
	*Time and material resources*
	Time
	Materials

**Fig. 2:**
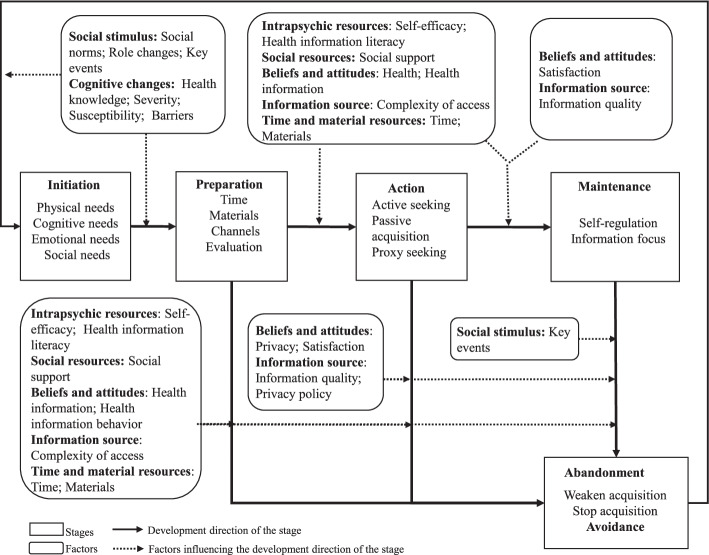
A theoretical framework of health information avoidance change

At the conclusion of this study, HIAC was understood as a non-linear heuristic process of 5 interrelated stages, affected by 7 factors. For ease of understanding, a graphical framework of the theory—the health information avoidance change model (HIACM)— is presented in Fig. 2.

With regard to the stages of HIAC, *initiation* (generating the need for change from avoidance to acquisition) is the starting point of psychological change. *Preparation* (preparing for the conditions necessary to acquire health information) and *action* (acquiring health information) are the intermediate stages that most changers go through. *Maintenance* (maintaining the health information acquisition behavior) is the desired outcome of change, but it is not a stage that every changer can enter. The last stage, *abandonment*, is the end (i.e., the failure) of a particular round of change. However, after a time, the changer may initiate a new round of change to obtain information and eventually bring about circular closure of the model.

The shifts among stages and the activities that individuals take in each stage are the consequences of the combined influence of various individual and environmental factors on the change. In addition to differences in sociodemographic characteristics, individual factors include cognitive change, belief and attitude, intrapsychic literacy, available social resources and time and material resources. Environmental factors mainly involve social stimulation and information source.

In the following subsections, the main categories of HIAC, their subcategories and their relation to the other main categories are described based on the analyzed interviews.

### Stages and related activities in HIAC

#### Initiation

At "*initiation*", the participants became aware of a demand to reduce or eliminate health information avoidance behavior; that is, motivation was generated to change the state of HIA. On contemplating the demand for change, physical, cognitive, emotional, and social needs emerged from the data.

Physical needs drove individuals to identify, prevent, and solve health problems by acquiring health information. In the interviews, all participants mentioned physical needs. For instance, Participant 8 (age between 26 and 45, in good health) stated: “*I felt that I was still young, and my physical fitness was good, no need to pay attention to it [fitness information].Of course, I don’t like sports, don’t want to exercise […] However, I found that my physical fitness has dropped significantly, need to improve […].*”

Cognitive needs are another important component of user information needs. Some participants expected to acquire health information to achieve goals such as accumulating health knowledge. For instance, Participant 9 (female, diabetic) mentioned: “*During that time [when she avoided health information], I never read books that tell you how to eat, never watched TV shows, or whatever. I didn’t want to watch or listen […] But there were always people who said that you couldn’t eat this, and you couldn’t eat that, [they] are sweet … I believed that diabetes does not mean that you cannot eat sweets at all. I wanted to learn whether experts thought so too […].*”

Emotional needs were also mentioned by all participants and which were mainly described as seeking psychological comfort and regulating negative emotions by acquiring health information consistent with their “expectations”.

Among social needs, impression management and obtaining a desired identity were most frequently mentioned by participants. Participant 3 (female, undergraduate degree), for example, stated: “*I’m already a mother, I can’t give my child a bad impression and let him think ‘my mother is afraid of doctors and going to hospitals’ […].*”

When demand for change reached a certain threshold under the combined effect of external social stimuli and internal cognitive changes, a shift from the initiation to the preparation stage took place. In some cases, the individual shifted directly to the action stage, without stopping at the psychological level.

#### Preparation

“*Preparation*” refers to the individual’s preparation for the subjective and objective conditions required for behavioral change. Planning time, preparing materials required to seek health information, seeking channels of health information that could meet their changing needs, and evaluating possibilities were the main activities in this stage.

Planning time included decisions regarding when to seek information and how often. Many participant s mentioned time management in advance of the change. They decided when and how often to obtain information, adjusting other daily activities according to the target information source service time window and the length of time it could take to obtain information.

Material preparation included purchasing smart devices, downloading apps, applying for medical cards, and so forth. For example, after deciding to seek professional doctors to help her prepare for pregnancy, Participant 13 (26–45 years old, postgraduate) described downloading the mobile apps for two hospitals “*I had been avoiding pregnancy-related information for about two years […] Later I felt I was of advanced maternal age, (she smiled), and it would be better to ask a doctor to help regulate my body […] I know that the hospital has apps*. *Experts’ outpatient information is available in the apps […] I installed the apps of two hospitals and started to search the expertise and outpatient information of the doctors I wanted.*”

In terms of information-seeking channels, interpersonal relationships, online search tools, and apps were most often mentioned. Participant 8 (26–45 years old, in good health) gave an example of associated preparatory action: *“I asked friends who like to keep fit for help and let them recommend fitness videos to me.*”

Evaluating possibilities mainly included evaluation of the availability of information and the benefits of change. In connection with the former, participants mentioned assessment of costs and possible social support; for the latter, solutions to health problems were most often mentioned.

#### Action

During the “*action*” stage, the individual started to take external actions to obtain health information, such as visiting professional institutions, interpersonal communication, accessing new media, searching for information on the Internet, and more traditional activities such as watching TV, listening to the radio, reading books, and attending lectures. Three types of activities were involved: active seeking, passive acquisition, and proxy seeking. Once clearly aware of their health information needs and able to express and act on those needs, changers usually adopted active seeking strategies, including through conversations with healthcare providers, family, and friends; web searches; accessing new media; and selecting TV shows.

In situations where the need was not clear or active seeking was not possible for a given participant, passive acquisition strategies such as waiting to encounter information were adopted. When, for various reasons, participants were unwilling to face the source of the information they wanted, they implemented proxy seeking strategies; that is, they entrusted others (e.g. children, friends, relatives) with helping them seek health information. For example, Participant 7 (≥ 61, suffered from acute stroke, junior college) reported: “*I didn't want to go to the hospital, because I feel nervous when I see doctors. […] So I asked my child to go to the hospital to ask the doctor for the check-up report.*”

#### Maintenance

“*Maintenance*” is characterized by having developed a habit of searching for or accessing target health information regularly. Self-regulation and information-focusing activities were often mentioned at this stage.

Even if some participants inevitably encountered various difficulties and obstacles, such as economic constraints, time conflicts, and skill barriers, they actively self-regulated through various means such as seeking help from others, self-encouragement, and scheduling in advance to maintain the acquisition state. For Participant 10 (male, corporate employee, undergraduate degree), this took the form of a daily habit: “*For a period of time, no matter when I went to bed, before going to bed, I would check if the WeChat public account [DingXiangYiSheng, a health information portal]) had been updated. Once it was updated, I would take a look. […] Sometimes I would forget when working overtime through the night, but the next day I would make up for it. […]*".

Information focuses were more obvious in the maintenance phase. The participants began to identify desired information sources or eliminate undesired information sources, and to access information sources selectively. For example, Participant 26 (26–45 years old, postgraduate) tried multiple pregnancy-related apps over a period of time but retained just one of them: “*Now there is only Mom Tree on my phone. I don’t have time to pay attention to all of them, and find it is not necessary to pay attention to everything.*”

#### Abandonment

"*Abandonment*" is the stage at which individuals abandon the change. At this stage they immediately stop acquiring health information, gradually weaken their acquisition behavior, or even return to actively avoiding health information.

A few interviewees chose to abandon their change after the preparation stage. Even if they had prepared time, materials, channels, and other objective conditions, they returned to the state of HIA because of, e.g., low self-efficacy, low health information literacy, or app and device operation issues. This was the case for Participant 16 (≥ 61, diabetic, high school and below): “*My son had installed diabetes management software for me. […] I haven’t learned how to operate it, so I just leave it aside, and have never used it. I don't want to use it anymore. It is too troublesome to use.*”

Several interviewees described giving up the change after the action stage. That is, they had already taken one or several health information acquisition actions. For example, Participant 14 (college student, in good health) installed a health tracking app and “*used it several times, mainly to see how long I slept at night and the changes in my heart rate […] but the experience was quite bad, the result seemed to be wrong, inaccurate, and [the app] was not easy to use. […] Anyway, I never used it after that.*”

There were also several interviewees who reported that they actively or passively stopped changing after maintaining their new behaviors for months or even years. The main manifestations included reduced frequency, shorter acquisition time, and conscious avoidance behavior, as in the case of Participant 12(male, 26–45 years old, chronic renal failure uremic stage, unmarried). This participant was “*no longer willing to tell the doctor about [his] condition,*” after an earlier search for health information: “*[…] for a few months, I kept looking for the uremia treatments information online […] still had to rely on dialysis […] Kidney transplantation is difficult and expensive[…] my father and mother had already borrowed a lot of money […]I don’t want to look for it anymore, just go for dialysis[…] I also don’t want to discuss it with the doctor. […]*”.

### Factors and their influences on HIAC

#### Cognitive change

Cognitive change refers to the change in an individual’s views, judgments, and cognitions about health, health information, and health information behavior. The participants’ cognitive changes included expanded health knowledge, changes in perceived severity of health problems, changes in perceived susceptibility to diseases, and changes in perception of barriers to health information acquisition, absorption, and utilization.

Only when the cognitive change reached a certain level could it drive a shift from demand to substantive behavioral change; otherwise, it might not progress beyond the initial stage. Participant 23 (46–60 years old, high school and below, retiree) at first refused any information about gastroscopy because of fear of pain, but after learning that gastroscopy could be performed painlessly, she began to ask her children to help find relevant information on the Internet: “*At the beginning, people told me about gastroscopy. I didn’t listen, or ask. I’m afraid of pain. Later, I happened to hear heard that there was a painless gastroscopy. People didn’t feel the pain. Then I went home and asked my son to go online to check if it was true.*”

#### Social stimulus

Also important were the social stimuli that prompted individuals to initiate changes and externalize them into actual behaviors. The social stimuli participants described included social norms, role changes and key events.

Social norms refer to the influence of the health information behavior patterns of other members of a specific group to which the changer belongs, especially those whom they respect, or with similar health problems or barriers to health information. One participant gave an instance of the impact of other members’ active health information searching behaviors on her own changes after participating in the community’s elderly chronic disease knowledge contest: “*I just said, I have diabetes, so they let me prepare the diabetes knowledge […]. I didn’t want to do it, especially in terms of food. But others were busy reading books, checking things online, consulting community doctors, and some even took notes and shared them with everyone in the WeChat group. Then I thought it would be bad for me to do nothing, and I couldn't hold back, so I started to ask my children to help me buy some books to read and prepare*” (Participant 11, 46–60 years old, diabetic, high school and below).

Role changes, as far as the description of the participants was concerned, mainly referred to the change of the individual’s role in the family and in organizations. For participants, those role changes could bring significantly different behavioral responsibilities that were more likely to promote the occurrence and maintenance of the change. Participant 3(female, undergraduate) explained that compared with being an aunt, being a mother had a greater effect on her changes: “*When I was not married, I lived with my younger brother. He also has children. I also helped take care of [his]) children […] Being a mother is different.*”

Key events refer to events that can influence individuals’ attention and time allocation, emotional changes, material security, etc., such as frequent business trips, examinations, privacy disclosures, and so on. Key events in the externalized change stages mainly exhibit a negative moderating effect. For example, after six months of maintenance, Participant 10(male, corporate employee, undergraduate degree) abandoned a change because of time pressure induced by frequent business trips: “*This habit lasted for about half a year. […] Later, I was busy at work and had to travel frequently, sometimes a week or two at a time. I didn't have time to pay attention to them, and gradually stopped reading those articles.*”

#### Beliefs and attitudes

Individuals also held various relevant beliefs and attitudes during the preparation, action and maintenance of HIAC; these include health beliefs, health information beliefs, health information behavior beliefs, privacy attitudes, and satisfaction.

If the participant had positive beliefs concerning health, health information, and health information behavior—that is, paying attention to health, believing that appropriate health information was beneficial to maintaining and promoting health, and believing that the acquisition of health information could help avoid negative health outcomes—then the change could proceed smoothly. In contrast, if the participant held negative beliefs, the change might be terminated at any stage. For instance, after attending several community-organized chronic disease self-management lectures, Participant 16 (≥ 61, diabetic, high school and below) concluded the talks were useless for diabetes control, so gave up the change midway through: “*I had listened to several lectures, but found it was meaningless and useless […] a waste of time, [I] never participated later.*”

Privacy attitudes manifested as individuals’ opinions on the protection of their information during health information acquisition or use. In the action and maintenance stage, the judgment of low protection and high leakage of personal information (health, behavior, identity, etc.) contributed significantly to abandonment of the change. Perceived requirements to over-authorize, unfair privacy policies, and lack of privacy controls appeared frequently in the data of interviewees, especially young participants. For example, Participant 26 (26–45 years old, postgraduate) said that “*every time I wanted to leave a message or ask a question, it made me register and complete a form. […] What did it need so much information for? […] Then I uninstalled [the apps].*”

Satisfaction here refers to the subjective evaluation of the degree of pleasure and satisfaction of health needs brought by the acquisition or use of health information. The objects of evaluation involved the quality of information content, software functions, information service quality, and health maintenance and promotion effects after information utilization. Satisfaction was related to the individual’s prior expectations and affected the individual’s choice of HIAC during the action and maintenance stages. If the relevant emotional experience did not meet psychological expectations, the change stopped. For example, Participant 7 (≥ 61, suffered from acute stroke, junior college) stopped observing changes in his blood glucose because he felt that his “smart” blood glucose meter did not live up to its name: “*I had used it several times, but I felt that it was not smart at all. It was different from what I thought: smart, it should be very simple, it shouldn’t be necessary for me to memorize the measurement results every day. It should record them itself and then show them to me. […] I stopped using it.*” Conversely, if the experience met or exceeded expectations, the interviewee might continue to seek health information. For example, because of satisfaction with the quality of the information content, Participant 5 (researcher, in good health, undergraduate degree) kept using a certain database to search the health information she needed: “*CBM is really authoritative, all the information I wanted can be retrieved, most of it. I am still using this database.*”

#### Intrapsychic resources

Intrapsychic resources points to the resources internal to the changer, here including self-efficacy and health information literacy.

Self-efficacy here means the degree of self-confidence to change. The higher the sense of self-efficacy, the more successful a person will be in entering the stage of action or maintenance; on the contrary, those with low self-efficacy are more likely to abandon the change. Sentences expressing low self-efficacy, such as remarks that one “cannot learn,” finds it “difficult to learn,” or “cannot persist,” frequently appeared in negative outcome data.

Health information literacy involves the intersection of health literacy and information literacy [61]. Participants with high health information literacy were more confident in changing their avoidance behavior; those with lower literacy found it more difficult to enter the action or maintenance stage. For example, when explaining the reason for her successful experience of internet health information avoidance behavior change, Participant 9 (diabetic, medical staff) mentioned: “*Fortunately, I still have some medical knowledge, simple things, I can still judge which are true and which are false. Otherwise, it was really impossible, [I] guess [I] would have given up [the change]*.”

#### Social resources

Social resources refer to social support that can be obtained through family, interpersonal relations, public services, etc.; these include informational, emotional, and economic support along with equipment, time, and technology. The provision of information could reduce the burden of information search and judgment and help participants get the health information they wanted faster and more accurately. The role of emotional support (or the lack thereof) was mainly to strengthen or weaken self-confidence in the change, as manifested mainly in verbal encouragement (e.g., “*you can*”) and negation (“*no use*”). Technical support could help solve the problem of insufficient information skills during the change, such as the struggles some elderly interviewees reported in learning to use health-related apps. Economic support, including the provision of equipment, was mainly described as helping solve the barriers of material conditions, such as “*recharge a payment plan online*” and “*buying smart phones*.” Time support meant providing sufficient free time to guarantee health information acquisition activities, e.g., “*The show started at 7 o’clock in the evening, usually right after dinner. I watched TV when it started, and my husband helped wash dishes*” (Participant 11, female, 46–60 years old, diabetic). The lack of external support could cause participants to choose to terminate the change. For example, Participant 20 (female, 46–60 years old, hypertensive, retiree) mentioned when explaining the reason for giving up a change halfway, “*Our children can drive, but they had to go to work, didn't have time to take us [to the hospital]*.”

In the interviews, children, teachers, friends, colleagues, communities, and schools were frequently mentioned by the interviewees as the actual main providers of social resources. Medical professionals were the social network that many participants expected to have in the process of change: “*I wanted to ask a doctor, but I didn't know anyone*,” “*It's great to have a nurse friend*.” This was mainly related to convenience, e.g., “*sav[ing] a lot of trouble*.”

#### Information source

The characteristics of the health information sources also play a role. When asked about what health information sources affected their behavioral choices (continue to change vs. abandon the change) and how, participants’ answers involved not only access to health information, but also the processing, acceptance, and utilization after information was accessed. Through contrasting examples provided by the participants, 3 information source characteristics were identified: complexity of access, information quality, and privacy policy.

Complexity of access refers to the degree of difficulty in acquiring information from health information sources (including equipment, software, people, and institutions). Operation (of software or equipment), time, economy, and communication were mentioned in varying degrees by different participants. For instance, when asked why they stopped visiting doctors, participants replied that “*registration*” was difficult; waiting time was too long; communication time was very short, “*only a few words*”; and communication was difficult (“*[I] don’t know what to ask, how to ask*”).

Information quality refers to various attributes that measure whether information content meets the needs of use. In the interview data, the most frequently mentioned attributes included the organizational framework, form of presentation, language of expression, release time, release organization, creator, consistency, advertising, and false information. These were related to subjective feelings and judgments of the information that the participants paid attention to: relevance (“*none of what I wanted*”), reliability (“*I usually only read the experts’ replies*”), practicality (“*too general*”), recency (“*The doctor’s information was several years ago, has not been updated*”), intelligibility (“*many medical professional words, I don’t understand*”), and interestingness (“*all text*”). Internet search engines were a highly accessible source of health information, but many participants reported that an excess of “*advertising and false information*” in the search results caused them to terminate their internet search behavior.

The privacy policy was expressed in terms related to privacy protection and was often mentioned among the negative outcomes following the action and maintenance stages. If participants make low privacy protection judgments based on the privacy policy, they tend to stop acquiring information from that source. Lack of privacy policy, simple terms, and perceived unfairness of terms are the main reasons for low privacy protection judgments. Participant 26 (26–45 years old, postgraduate) described the negative effect of the privacy policy as follows: “*Those terms are too simple, almost the same as if there are no terms. It's useless if something happens really. […] Then I uninstalled [the apps]*.”

#### Time and material resources

Health information avoidance behavior change also depends on the time an individual can allocate to the change and the material conditions in which the change takes place. Time denotes whether the participant had enough time to implement or complete the change. Materials include money, transportation, equipment, and other material elements needed for the change.

Time and materials constituted the basic conditions of the change behavior, which moderated the speed and likelihood of transitioning between stages after the change was initiated. If the participant had the requisite time and materials, they were more likely to continue the change; otherwise, they tended to terminate the change. For example, Participant 13 (26–45 years old, postgraduate) had considered giving up due to financial issues: “*[…] at that time, we had just bought a house, and we had little money, but an inspection fee cost several hundred [yuan], [so I] had thought about giving up [the change].*”

## Discussion

### Principal findings

#### Stages and activities

As in previous research on the process of information behavior from a nonlinear perspective [46–48], the stages and shifts described by the model show change from health information avoidance to health information acquisition to be multi-stage, non-linear, and heuristic. Initiation, preparation, action, maintenance, and abandonment are the five core stages of HIAC. Maintenance is the desired outcome of change, but an individual may shift to abandonment at any intermediate stage, which means the failure of this round of change. Unlike the stage variables in TTM [62], which were refined and defined comprehensively based on time, psychology, and behavior, the stage variables in HIACM were inducted based on the latter two. This is because participants in this study did not indicate that the change had obvious length-of-time characteristics, i.e., describe how long they stayed or planned to stay in each stage. HIAC is also an iterative process. After entering the last stage of abandonment, the changer may initiate a new round of change, at which point the changer may stay in each stage for a very short time or even “omit” some stages.

Consistent with existing literature [63], the information acquisition activities in the action stage can be summarized into three types: active acquisition, passive acquisition, and proxy acquisition. The choice of activities is related to several factors. In addition to being limited by medical knowledge and new media literacy [64], unpleasant emotional experiences and the anticipation of a complexity operation would also prompt people to choose a proxy strategy. This study also found that the corresponding results seem to be somehow related to the choice of strategy. In the cases reported by the participants, changes guided by the active strategy have obvious characteristics of initiative and purpose, and were more likely to develop to the maintenance stage. Because of worries about troubling and burdening others, only 2 of the 5 participants who adopted proxy acquisition as the main change strategy entered the maintenance stage.

Compared with the non-directional health information acquisition in the action stage, the information focus in the maintenance stage was very significant; that is, participants focused on specific health information sources. In addition to the lack of sufficient time mentioned by participants, the reason may also be related to changing motivation [65], health information acquisition intention and tasks [66, 67], and the order of the individual’s daily life as noted in the Savolainen Model [68].

#### Factors and their association with stages

The change process is affected by multiple factors, and the effects of these factors at different stages seem to vary to a certain extent, though it is difficult to define which factor(s) are playing a major role in a specific stage with our study design.

Cognitive changes and social stimulus mainly had an impact during the initiation phase of the change, promoting the shift from inner psychological activity to external behaviors. The finding here that cognitive changes might activate intention to change is somewhat consistent with the previous findings that beliefs and attitudes influence health information seeking behavioral intentions. However, in our interviews, the participants emphasized the “changes” in opinions and judgments, that is, changes in perceived severity of health problems, changes in perceived susceptibility to diseases, and changes in perception of barriers to health information acquisition, absorption, and utilization. Therefore, ultimately we opted for “cognitive change.” As a part of social stimulus, social norms’ positive effects on the change are in line with the findings of Taylor and colleagues [69].

Beliefs and attitudes about health information and health information behaviors, intrapsychic self-efficacy and health information literacy, social resources, complexity of information access, and the availability of time and materials all had an impact on the development of the entire outer change process. These have been corroborated by health information circumvention and search studies. For example, a review [70] has shown that low self-efficacy and health information literacy are both related to avoiding health information; while there are strong evidences that high self-efficacy and health information literacy are related to seeking behaviors [71, 72].With regard to social resources, social support has been found to significantly influence people’s intentions to seek [73] or avoid health information. Time and materials constituted the basic conditions of day-to-day life information seeking [68].

Privacy attitudes and satisfaction with the information quality and privacy policy of information sources began to play a significant role after entering the action stage. Key events mainly started to take effect during maintenance. Moreover, privacy attitudes, privacy policies, and key events primarily encourage the negative development of change; these factors appeared in many participants’ explanations of the reasons for giving up change, but rarely appeared in descriptions of the reasons for positive outcomes. In fact, some studies have shown that in the environment of network and social media penetration, privacy attitudes have become a hindrance to initial and ongoing information acquisition [74, 75]. The greater the individual's concern about privacy, the more sensitive they are to privacy policies, and accordingly, the greater the impact of privacy policies on their health information behavior [76].

Furthermore, young participants in the present study mentioned “privacy” more frequently and seem paid more attention to it. Age and gender are not significantly associated with other factors. For example, participants across age groups mentioned their desire to receive social support to varying degrees; the younger ones hoped to receive this support in the form of medical knowledge, and the older ones hoped to receive assistance with information technology.

#### Theoretical framework

The third aim of this study was to relate the stages and factors included in HIAC to form a theoretical framework that deeply illustrates the process of change. The existing controlled experimental research on reducing health information avoidance intention or behavior [41, 42, 77] was mostly conducted from the perspective of inducing factors, with less consideration to the procedural characteristics of behavior change from the thoughts and experiences of the avoider. Consequently, to our knowledge, no relevant theoretical framework or model has been constructed. Our framework contributes to addressing the process of change by integrating stages and factors, although in the real world this process may sometimes be very short-lived.

We argued that the change from health information avoidance to acquisition is a type of health behavior change. There are, in fact, theories present the stages of such a change and their relationships: TTM [43, 44], one of the popular health behavior change theories, was a great inspiration for this research. However, apart from the different phases and their naming and definitions, the relationship among stages in HIAC was not sequential as shown in TTM [45] but heuristic. After generating the motivation to change, individuals may move to “*abandonment*” of the change from any prior stages. As far as our interview data is concerned, individuals with strong motivation and the conditions for obtaining health information (such as high network health information literacy) could quickly enter the *preparation* and/or *action* stages. Furthermore, HIACM does not end with a successful “termination” as in TTM or ideal “*maintenance*” in our analysis, but with “*abandonment*”, which represents a failure to change. At “abandonment”, one type of health information behavior is avoidance, or returning to the avoidance state, which logically constitutes a closed loop, further indicating the non-linear characteristics of the health information behavior change.

The heuristic showed in HIACM is in line with models on information seeking behavior, such as Foster’s nonlinear model [47]. Our framework helps to address the heuristic patterns of individuals’ change from avoiding to seeking health information. Wilson’s model [54] of information behavior can offer possibilities for explaining why change does not go smoothly by integrating risk/reward theory and self-efficacy, but it is too abstract for the present purpose because of its focus on macro-behavior. To explain the heuristic characteristics of HIAC more clearly, we also strived to distinguish which factors effected the positive and negative development of change respectively, and to reflect both in HIACM. Specifically, following the developmental path of change, we analyzed the influencing factors of each transition between stages. As shown in Fig. 2, the set of factors at different stages changed dynamically and the positive and negative promoting effects of these factors were not completely consistent.

### Implications

#### Implications for research

The change from health information avoidance to health information acquisition should be regarded as a subcategory of health information behavior. Aside from the pre-factor of “avoidance,” it may seem similar to health information seeking behavior, but just as avoiding information is not the same as non-seeking and selection [2], it is precisely because of this pre-factor that the distinction must be made. Therefore, existing health information seeking behavior models cannot be directly used to explain this change process. Inspired by behavior change theories [43, 62] and non-linear information seeking behavior theories [46, 47], this study paid close attention to the process of change from health information avoidance to acquisition. Five core stages of the change and related factors that affect their relationships were identified from qualitative empirical data and were integrated to form a grounded theoretical framework, which can be offered as means of more deeply r understanding individuals’ change from health information avoidance to acquisition. Furthermore, this framework can provide a reference for subsequent qualitative or quantitative research on HIAC: for instance, in the selection of research questions.

For us, the development of HIACM provides an in-depth understanding of consumers’ health information behaviors, and helps researchers establish a new knowledge link between health information avoidance and health information acquisition. This may lead to further research endeavors. For example, the discovery of stage variables and factor variables builds foundational knowledge and provides us with new insights into comparative research on health information behavioral interventions; the extracted concepts and corresponding original descriptive texts can help us develop more understandable questionnaires or follow-up interview studies.

#### Implications for practice

The results showed that change from health information avoidance to health information acquisition was a dynamic process with multi-faceted influencing factors. Avoiders and interventionists should not always treat the change as a short-term or single event, even though one or several stages may arise very quickly or abruptly in the actual change process. Moreover, the entire change process cannot always be completed smoothly. Even in the case of an ultimately successful change, the changer may have been stuck at a certain stage for a long time, or even fall back to the previous state. Interventionists must observe changers’ behavior to identify their probable current stage, then use a dynamic and comprehensive perspective to judge related obstacles or facilitating factors and adopt targeted interventions to promote the change.

As far as responsible subjects involved in influencing factors are concerned, HIAC is not only an individual but also a social behavior. It requires the collaboration of individuals, families, health information providers, healthcare providers, and governments. For the individual, the focus is to solve one’s lack of intrapersonal resources by actively participating in health information literacy training, changing unreasonable health beliefs, establishing correct health information beliefs, and enhancing the self-efficacy of change (e.g., by learning from seekers who share common characteristics with oneself). The role of families in the process is to provide social support including, e.g., emotional encouragement, information technologies and professional knowledge related to health information acquisition, and the time and economic resources required for the change. For health information providers, the focus is on ensuring the quality of health information (especially understandability and practicality); reducing the complexity and time cost of health information acquisition (such as by providing self-contained and contextualized voice or video explanations); and protecting the privacy of individuals’ identities and health information behaviors. Healthcare providers have greater advantages in enacting personalized health information interventions [78]. They can consciously transmit understandable health information as they provide daily diagnosis and treatment, follow-up, examination, and other health services. Apart from public health services and medical insurance [27, 30], governments can provide policy-level support for HIA intervention by creating multi-level public health information literacy education plans and strengthening privacy management in health information services.

In addition, the finding that the influencing factors are not completely consistent in their effect must be noted by interventionists: individuals vary in their sensitivity to the lack of similar factors in positive acquisition behavior and negative avoidance behavior. This may imply that the lack of facilitating factors needed for positive health information behavior may prompt changers to turn to negative behavior quickly, but resolution of the triggering factors of negative health information behavior may not in itself lead to changes in a positive direction. Further, privacy-related factors and key events seem to play a role mainly in negative development of the change; these have also been shown to be related to health information avoidance behavior in general [2, 4, 5]. Therefore, interventionists should pay sufficient attention to privacy protection during the intervention, and the intervention should be implemented to avoid, as far as possible, the period when adverse key events may occur.

Ultimately, we also believe that the HIACM model is inspiring and helpful for promotion of health information seeking irrespective of whether the person originally avoided health information. The actions that follow from a decision to obtain health information are not all one-time events; the factors included in HIACM can also affect the acquisition process and even trigger the emergence of avoidance behaviors.

#### Limitations and future research

It was expected that the emerging theoretical framework would be applicable across different contexts of change from health information avoidance to health information acquisition. However, this study had the following limitations: firstly, the theoretical sampling in this study did not specifically distinguish the avoidance motivation, the type of avoidance, the type of health information, and the duration of avoidance state prior to the change; secondly, the local language ability of the researchers limited the sampling of rural areas; thirdly, the interviews with non-local samples were conducted in a non-face-to-face manner, making it impossible to observe non-verbal information such as expressions and actions. These may lead to a certain deviation between the analysis results and reality; incorporating further research results with heterogeneous samples would improve the stability of the model. Fourthly, in a strict sense, HIACM is still in the pre-theoretical stage; it may be described as an open framework for thinking about the change from health information avoidance to acquisition. Therefore, improving the theoretical level of the model is another important direction for future efforts.

Moreover, in keeping with grounded theory, this study attempted to identify which factors play a role in which stages, but it was difficult to distinguish the degree of effect for related influencing factors at each stage. In the future, it is necessary to develop measurement scales based on the concepts involved in the model, to assess the redundancy and significance of the theoretical components in the model through quantitative research, and to analyze the effect of sociodemographic characteristics on variables in HIACM. Such work will yield a more practical model, both in general and in specific contexts including avoidance motivation, type and topic of health information, and individual differences (e.g. geography, gender, age, education, income, health history, health information literacy, and personality traits).

## Conclusions

This paper contributes to the understanding of health information behavior and behavior change by investigating why and how individuals change from avoiding health information to acquiring health information and by developing a relevant information avoidance behavior change model, HIACM. HIACM illustrates this process of change in a way that reflects the experience of the changers. The names given to the stages—initiation, preparation, action, maintenance, and abandonment—almost suggest a sequence of activity. However, the shift between stages described in HIACM show the change to be nonlinear and holistic, taking into account individuals’ cognitive changes, social stimulus, beliefs and attitudes, intrapsychic literacy, available social resources, information source, and other situational factors. We believe that HIACM is capable of guiding intervention design for avoiders in different stages of change. Designers of both self-interventions and external interventions are encouraged to consider the multi-stage, non-linear, and holistic character of the change process, and to pay more attention to the factors that may hinder change as informed by HIACM.

## Supplementary Information

**Additional file 1.** Initial sample semi-structured interviewguideline.

**Additional file 2.** Sociodemographic questionnaire.

**Additional file 3.** Codebook of subcategories: name, examples of open concepts, definition,and category.

## Data Availability

The datasets used and/or analyzed during the current study are available from the corresponding author on reasonable request. The data contain information that could compromise research participant privacy, and making the data publicly available would violate the terms of their consent.

## Abbreviations

GT: Grounded theory
HIA: Health information avoidance
HIAC: Health information avoidance change
HIACM: Health information avoidance change model
SGT: Straussian grounded theory
TS: Theoretical sampling
TTM: The transtheoretical model
TV: Television
Apps: Applications
CBM: Chinese BioMedical Literature Database
