# Supplementary material for: Developing a framework for understanding health information behavior change from avoidance to acquisition: a grounded theory exploration
Source: BMC Public Health. 2022 Jun 4;22:1115. doi: 10.1186/s12889-022-13522-0 (PMC9166210; doi:10.1186/s12889-022-13522-0)
Supplement: Supplementary file 1 — Additional file 1. Initial sample semi-structured interviewguideline. [file 12889_2022_13522_MOESM1_ESM.doc]

**Initial Sample Semi-Structured Interview Guideline**

## Introduction (5 mins)

The researcher performs a self-introduction, goes over the consent information with the participant, introduces the whole interview schedule, and emphasizes again: “*At any point, for any reason, you are welcome to take a break or stop the interview altogether*.”

## Concept explanation or confirmation (5 mins)

The researcher re-explains or answers questions about the core concepts involved in this research: health information, health information acquisition, and health information avoidance.

## Opening Questions (30 mins)

***Tips:***

- *Except for the initial question, all questions are optional, and there is no fixed order, depending on the interviewee’s* *narration or answers.*
- *Suggest that the participant add the following to the timeline: thinking and turning points.*
- *Further questions* *arise from the answers.*
- *After the participant’s initial narration is done, ask him/her some questions to try to clarify points that may be unclear, if any, on the timeline.*

### Initial questions

- Could you tell me how you experienced change from avoiding health information to seeking health information? One or more stories.

### Some possible probes

- Why do you want to make the change?
- Have you ever wanted to give up, or given up, during this period? Could you tell me why?
- Was there any difference during the whole change period? If yes, could you tell me about the differences?
- Could you tell me about what happened next?
- Do you think this process went smoothly? And could you tell me why?
- Are there any differences in the reasons why you choose to continue or terminate the changes during this process? If yes, could you tell me what they are?
- How long did this state or phase last?
- Why didn’t you continue …?
- How did you feel and what did you think when …?
- How did it affect your choice?
- What did … mean to you at that time?
- What was the situation at that time?
- What was more important to you at that time?
- If this factor was different, would you change your choices? If yes, could you tell me why?
- Is there any other reason? If yes, could you tell me what they are?

### Concluding questions

- Is there anything else you want to tell me?
- Is there a different experience? If so, tell me what is different?
- Do you have anything to ask me?

## Background survey (5 mins)

The researcher asks the participant to fill in the background questionnaire to learn his/her population and social characteristics and answers questions that the participant may encounter.

## Conclude the interview (5 mins)

The researcher thanks the participant, invites the participant to recommend other interviewees and concludes the interview.
