# Supplementary material for: Developing a framework for understanding health information behavior change from avoidance to acquisition: a grounded theory exploration
Source: BMC Public Health. 2022 Jun 4;22:1115. doi: 10.1186/s12889-022-13522-0 (PMC9166210; doi:10.1186/s12889-022-13522-0)
Supplement: Supplementary file 2 — Additional file 2. Sociodemographic questionnaire. [file 12889_2022_13522_MOESM2_ESM.doc]

**Sociodemographic questionnaire**

**Dear friends:**

Thank you very much for accepting and participating in this interview! Now we need to learn some sociodemographic information about you. Please fill out this questionnaire according to the actual situation. If you have any questions or need help, please feel free to let me know.

Note: 1) Unless otherwise specified, only one answer item can be selected; 2) If you choose the "Other" option, please give a brief explanation, either orally or in writing.

**Thank you again for your support and cooperation!**

1. Your gender:

2. Your age: years

3. Your education level is: ( )

**A**. No formal education **B**. Junior high school **C**. High school
**D**. Technical secondary school **E**. Junior college **F**. Undergraduate **G**. Postgraduate

4. Monthly disposable income per capita of your family: ( ) *【RMB: Yuan】*

**A**. <1000 **B.**1000 to 2000 **C.**2001 to 3000 **D**.3001 to 5000 **E**. > 5000

5. Medical insurance you have participated in: ( )

*Note: You can choose more than one.*

**A**. None **B**. Urban residents' basic medical insurance **C**. Urban employee medical insurance **D**. New rural cooperative medical insurance **E**. Major illness overall planning **F**. Public-funded medical **G**. Commercial insurance **H**. Self-funded medical **J**. Other

6. Your marital status: ( )

**A**. Unmarried **B**. Married **C**. Widowed **D**. Divorced **E**. Other

7. Your place of residence: ( )

**A**. Urban **B**. Rural **C**. Other

8. Who lived with you during the change? ( )

*Note: You can choose more than one.*

**A**. Spouse **B**. Children **C**. Grandchildren **D**. Parents **E**. Colleagues **F**. Classmates **G**. Friends **H.** Living alone **J.** Other

9. Your current occupation:

*Such as: business owner; student; farmer; teacher; doctor; nurse; retiree; unemployed, etc.*

10. Your employer:

*Note: If you filled in “unemployed” in question 9, you don't need to answer this question*

11. Your current health status: ( )

**A.** Good **B**. Suffer from chronic disease **C**. Suffer from major disease **D**. Other

12. Your health history: ( )

**A.** Has been good **B**. Has had chronic disease **C**. Has had major disease **D**. Other
