# Supplementary material for: Developing a framework for understanding health information behavior change from avoidance to acquisition: a grounded theory exploration
Source: BMC Public Health. 2022 Jun 4;22:1115. doi: 10.1186/s12889-022-13522-0 (PMC9166210; doi:10.1186/s12889-022-13522-0)
Supplement: Supplementary file 3 — Additional file 3. Codebook of subcategories: name, examples of open concepts, definition,and category. [file 12889_2022_13522_MOESM3_ESM.doc]

# Codebook of Subcategories: name, examples of open concepts, definition, and category

| **No.** | **Name** | **Examples of concepts** | **Definition** | **Category** |
| --- | --- | --- | --- | --- |
| 1 | Physical needs | Disease prevention; Disease therapy; Strengthening physique | The individuals’ needs to maintain or promote physical health. | Initiation |
| 2 | Cognitive needs | Accumulating health knowledge; Learning skills | The individuals’ needs to perform health-related tasks that require cognitive effort. | Initiation |
| 3 | Emotional needs | Seeking psychological comfort; Regulating emotions | The individuals’ needs to maintain emotions such as pleasure and enjoyment. | Initiation |
| 4 | Social needs | Impression management; Status identity; Sense of belonging | The individuals’ sociality needs such as management of impressions, the pursuit of a specific identity and the sense of belonging. | Initiation |
| 5 | Planning time | Available time analysis; Scheduling time; Defining acquisition frequency | The individuals take actions in advance to meet the time or frequency required for change. | Preparation |
| 6 | Preparing materials | Purchasing smart devices; Installing software; Handling medical cards | The individuals take actions in order to meet the material conditions required for obtaining health information. | Preparation |
| 7 | Seeking channels | Seeking internet tools; Seeking professionals; Seeking professional institutions | The individuals take actions to identify sources and access methods for the health information that they want. | Preparation |
| 8 | Evaluation | Evaluating possibilities; Evaluating financial cost; Assessing time cost | The individuals evaluate the availability, cost, and benefit of the information. | Preparation |
| 9 | Active seeking | Active questioning; Online retrieval; Selecting information | Initiative and purposeful information acquisition activities. | Action |
| 10 | Passive acquisition | Waiting for information push; Encountering information | Activities that involve accidentally encountering information or obtaining it without demand. | Action |
| 11 | Proxy seeking | Entrust children with obtaining; Entrust friends with consulting; Entrust nurse with searching | Acts of entrusting others with the task of obtaining information. | Action |
| 12 | Self-regulation | Self-encouragement; Self-remedy; Scheduling in advance | The individuals actively adjust their mindset and behavior to maintain health information acquisition habits. | Maintenance |
| 13 | Information focus | Information theme focus; Information source focus; Information media focus | The individuals consciously lock information within a specific range. | Maintenance |
| 14 | Weaken acquisition | Frequency reduction; Weak interaction; Occasional browsing | The frequency and quality of health information acquisition behaviors are gradually decreasing. | Abandonment |
| 15 | Stop acquisition | Stop consulting; Cancel subscription; No longer online; Uninstall software | Completely stop any health information acquisition activities. | Abandonment |
| 16 | Health information avoidance | Self-concealment; Ignoring information; Denying information | Any behavior intended to prevent or delay the acquisition of available but potentially unwanted information. | Abandonment |
| 17 | Role changes | Family role change; Organizational role change | The role of the individual in the family or organization has changed. | Social stimuli |
| 18 | Social norms | Community norms; Mentor example; Leader demonstration | A customary pattern or code of conduct shared by members of a particular group to which the individual belongs. | Social stimuli |
| 19 | Key events | Long-term business trip; Examination; Privacy disclosure | Events that can provoke a change in individuals’ attention, time allocation, emotions, etc. | Social stimuli |
| 20 | Expanded health knowledge | Network knowledge; Disease knowledge; Surgical knowledge | The individuals’ knowledge system is expanded. | Cognitive change |
| 21 | Change in perception of barrier | Changes in perceived technical barriers; Changes in perceived communication barriers; Transportation assistance | The individuals perceive that the barrier to change has become weak enough to cope with. | Cognitive change |
| 22 | Change in perceived severity | Perceived symptoms became severe; Pain diminished | The individuals’ perception of the severity of health problems has changed. | Cognitive change |
| 23 | Change in perceived susceptibility | Feeling more susceptible to illness | The Individuals’ perception of their possibility of contracting a disease has changed. | Cognitive change |
| 24 | Health beliefs | Self-healing; Preventable; Unpreventable | The factual views, opinions, and judgments held by the individuals regarding health and health issues. | Beliefs and attitudes |
| 25 | Health information beliefs | Useful; Helpful; Available | The factual views, opinions, and judgments held by the individuals on health information. | Beliefs and attitudes |
| 26 | Health information behavior beliefs | No difference; Useless work; Hard to say; Burden | The factual views, opinions, and judgments held by the individuals on health information acquisition actions. | Beliefs and attitudes |
| 27 | Satisfaction | Wasting time; Unusable; Poor usability | The individuals’ subjective evaluation of the benefits after the health needs or health information needs are met. | Beliefs and attitudes |
| 28 | Privacy attitudes | Privacy protection; Privacy concerns | The individuals’ views on the security and protection of personal information. | Beliefs and attitudes |
| 29 | Self-efficacy | Hard to insist; Self-confidence; Diffidence | The individuals’ self-confidence in changing health information avoidance behavior. | Intrapsychic resources |
| 30 | Health information literacy | Health knowledge; Expression of information requirement; Information retrieval ability | A series of abilities including awareness of health information needs and ability to obtain, evaluate, analyze, and understand health information for reasonable health decision-making. | Intrapsychic resources |
| 31 | Social support | Family support; School support; College support | The care and help obtained from society, including but not limited to encouragement, materials, information, and services. | Social resources |
| 32 | Complexity of access | Complex process; Ease of navigation; Obscure labels | Operational complexity of information acquisition tools or media. | Information source |
| 33 | Information quality | Unreliability; Difficult to operate; Easy to understand | A collection of various attributes used to measure whether the information meets users’ needs. | Information source |
| 34 | Privacy policy | Privacy protocol; Registration protocol; Terms of agreement | Regulations or action guidelines related to users’ personal information security and protection. | Information source |
| 35 | Time | Free time; Lack of time; Available-time uncertainty | The time that can be freely allocated to improve health information behavior. | Time and material resources |
| 36 | Materials | Money; Smart phones; Network | The economic, equipment, transportation, and other material conditions required for a change in health information avoidance behavior. | Time and material resources |
